# Living Matter Observations with a Novel Hyperspectral Supercontinuum Confocal Microscope for VIS to Near-IR Reflectance Spectroscopy

**DOI:** 10.3390/s131114523

**Published:** 2013-10-25

**Authors:** Francesca R. Bertani, Luisa Ferrari, Valentina Mussi, Elisabetta Botti, Antonio Costanzo, Stefano Selci

**Affiliations:** 1 Istituto dei Sistemi Complessi, Consiglio Nazionale delle Ricerche, Via del Fosso del Cavaliere 100, Rome 00133, Italy; E-Mails: francesca.bertani@isc.cnr.it (F.R.B.); luisa.ferrari@isc.cnr.it (L.F.); valentina.mussi@isc.cnr.it (V.M.); 2 Dermatology Department, University of Tor Vergata, Viale Oxford 81, Rome 00133, Italy; E-Mail: elisabetta.botti@uniroma2.it; 3 Dermatology Unit-NESMOS Department, Sapienza University of Rome, Sant'Andrea Hospital, Via di Grottarossa 1035, Rome 00189, Italy; E-Mail: antonio.costanzo@uniroma2.it

**Keywords:** confocal, hyperspectral imaging, spectroscopy, 87.64.mk, 78.40.-q, 87.64.km, 78.30.Er, 02.50.Sk

## Abstract

A broad range hyper-spectroscopic microscope fed by a supercontinuum laser source and equipped with an almost achromatic optical layout is illustrated with detailed explanations of the design, implementation and data. The real novelty of this instrument, a confocal spectroscopic microscope capable of recording high resolution reflectance data in the VIS-IR spectral range from about 500 nm to 2.5 μm wavelengths, is the possibility of acquiring spectral data at every physical point as defined by lateral coordinates, X and Y, as well as at a depth coordinate, Z, as obtained by the confocal optical sectioning advantage. With this apparatus we collect each single scanning point as a whole spectrum by combining two linear spectral detector arrays, one CCD for the visible range, and one InGaAs infrared array, simultaneously available at the sensor output channel of the home made instrument. This microscope has been developed for biomedical analysis of human skin and other similar applications. Results are shown illustrating the technical performances of the instrument and the capability in extracting information about the composition and the structure of different parts or compartments in biological samples as well as in solid statematter. A complete spectroscopic fingerprinting of samples at microscopic level is shown possible by using statistical analysis on raw data or analytical reflectance models based on Abelés matrix transfer methods.

## 1. Introduction

Since the introduction of the basic confocal principle in 1955 [1], Laser Scanning Confocal Microscopy (LSCM) has rapidly become an effective and necessary scientific instrument for the observation of small structures in many fields, from material to life science, and is nowadays widely applied by biomedical researchers. The confocal system uses spatial filtering to eliminate the out-of-focal-plane light, thus gaining high spatial resolution and signal-to-noise ratio, and has the capability of vertical optical sectioning, which enables three dimensional imaging of thick samples.

Among the applications of confocal microscopy, fluorescence methods are the most widespread ones, also thanks to the increasing rapid development and commercial availability of fluorescent dyes and markers.

The current need to use and image multiple fluorescent labeling tags simultaneously, to discriminate different fluorescent components, and implement more complex functional experiments, such as Förster Resonance Energy Transfer (FRET) [2], Fluorescence Recovery After Photobleaching (FRAP) [3], Fluorescence Lifetime Imaging Microscopy (FLIM) [4] and Fluorescence Correlation Spectroscopy (FCS) [5], prompted the development of instruments able to perform fast multispectral confocal mapping. The goal is to merge sufficient spatial, spectral, and temporal resolution in order to collect a more complete picture of the sample, especially when dealing with live specimens.

Besides fluorescence, spectral information in confocal microscopy can also be obtained through different techniques such as absorption, reflection, transmission, emission, photoluminescence or Raman spectroscopy. Among various methods, the reflectance approach has recently gained great consideration [6,7], especially in the field of dermatology diagnostics, even if in most cases it is not associated to a fully spectral concept, but rather to a multimodal technique [8,9]. Differently, Raman spectroscopy can be used as a probe of the vibrational energy levels within a molecule, thus enabling the generation of a detailed chemical map of the sample [10].

The extension of the spectral range to the infrared is particularly important for biological samples, such as cells and tissues, in order to gain information regarding cellular components and metabolic status, and increase the depth of the analysis. In fact, IR-based techniques, both in the near- and mid-infrared regions, have shown huge potential in biomedicine because, being reagent free, they can rapidly and noninvasively detect changes in the biochemical composition of cells and tissues. FTIR micro-spectroscopy has been successfully applied to distinguish between different tissue types [11] and between the states of health, maturation, and differentiation of cells, with interesting applications for diagnostic purposes at clinical levels [12–14]. However, IR spectroscopy and spectro-microscopy have limited spatial resolution and limited time resolution, due to the slow acquisition time, or require the use of synchrotron radiation as a bright source [15].

In this context, Hyperspectral Imaging (HSI), also known as Chemical or Spectroscopic Imaging, has emerged as a powerful technique that integrates conventional imaging and spectroscopy [16,17]. Its introduction as a specific feature of LSCM, following the development of white laser sources, is quite recent and not common, and allows one to greatly enhance the performance and flexibility of the microscope [18]. Unlike a typical reflectance confocal image, a hyperspectral image provides a complete spectrum of the sample at every pixel location, so that, in a confocal configuration, one can obtain 3D morphological features together with local spectral information.

Here we describe a novel confocal microscope aimed at integrating structural and morphological information with detailed spatially resolved spectroscopic properties, powered by a supercontinuum laser source in the visible and near infrared spectral ranges, and able to collect a complete spectroscopic image by the acquisition of a one-shot wide range reflectance spectrum for every image pixel in the three dimensional sets of data typical of a confocal microscope. The microscope has an achromatic light path which guarantees that each wavelength contribution is effectively related to the same point of the sample.

The microscope performance is shown here in detail including axial resolution, achromaticity, lateral resolution, as well as the potential applications in different research areas as a powerful technique for *in situ* real-time imaging and spectroscopic investigation. We prove how the hyperspectral confocal microscope provides structural and chemical analysis of surfaces and interfaces in multilayer materials. Furthermore, we report our results of hyperspectral reflectance analysis of visible and NIR radiation from human primary melanoma cells in culture. We will show that differences between spectra from cytoplasmic areas and nuclear areas allow distinguishing cellular compartments without any exogenous dye and using a multivariate analysis approach. The application of our microscope to the analysis of scattering and heterogeneous tissue samples is under investigation. Preliminary results show that it is possible to obtain images very similar to those acquired with commercial reflectance confocal microscopes used for dermatologic applications working with selectable laser lines. The spectroscopic analysis of tissues results is under development and will be the argument of our subsequent experimental activity.

## 2. Concept and Technical Description

The confocal microscope presented here has been designed and realized by our group with a strong focus on the spectroscopy side. The instrument is completely conceived and realized from scratch to provide a versatile laboratory microscope able to explore different possibilities about the accessible spectral ranges, optical insertions, beam manipulation and sample access. The underlying idea is to realize simplified versions of the microscope to eventually address specific classes of samples for clinical or industrial applications.

A preliminary set-up and results have been previously reported [18]. Since then, the electronic layout has been completely changed, and the full IR exploitation has been completed with the insertion of a suitable array detector. Therefore, we are now able to completely report about the axial and lateral performances in terms of resolution and achromaticity.

**Figure 1.**
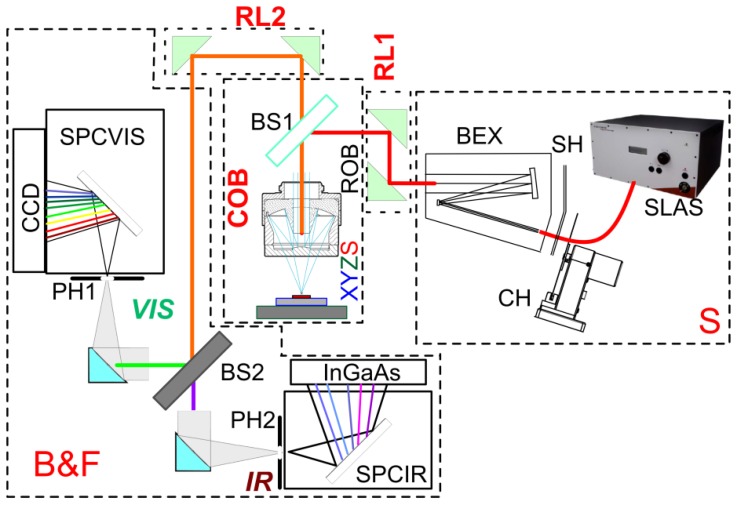
Microscope layout and the optical pathway. Abbreviations are given in the text.

The following section describes the current set-up. Then, a discussion is made about the optical properties of the most relevant parts. Following the optical path described in Figure 1, from right to left, we see different optical blocks. The source block S includes: the supercontinuum laser (SLAS; NKT SuperK Power+, NKT Photonics, Birkerød, Denmark), a unit able to deliver on average up to 1 mW/nm over the 0.5 μm–2.4 μm optical range, a beam shutter (SH; SR474 by Stanford Research Systems, Sunnyvale, CA, USA), a chopper (CH; Thorlabs 2000 crystal-stabilized unit, Newton, NJ, USA), and two coupled all-reflective beam-expanders (BEX; units BE02R/BE04R by Thorlabs), capable of overall achromatic reflectance >90% between the 0.5 μm and 20 μm wavelengths. The beam expander is needed to fill completely the objective entrance: the fiber output of the supercontinuum laser has, in fact, a fitted collimator with an output beam diameter of about 1 mm at 800 nm, actually depending linearly on the wavelength, and a divergence <0.5 mrad, unable to fill the objective input, as we will see later in the following section. The relay optics (RL1) section, which is only assembled with plane mirrors, is placed just after the source block. This section is used for beam alignment and to diaphragm the beam by additional iris; moreover, a moveable mirror can insert into the optical path another more conventional laser, to carry out, for instance, fluorescence measurements.

The condenser/objective block (COB) is formed by only two components carefully aligned on the beam path: the beam splitter (BS1) and the reflective objective (ROB). We have available many different laminas to be used as beam splitters. Different materials and/or thicknesses have been tested to minimize spurious effects and modulate the power fed onto the sample. For the current application, we have determined that a Borosilicate Crown glass (BK7) lamina of 160 μm thickness is the best suited. The reflective objective is a reverse Cassegrain following the Schwarzschild design (50102-02 model, Newport, Irvine, CA, USA), with 36× magnification and a back focal length at infinity. The reflective aluminum internal coating is over coated with magnesium fluoride (MgF_2_) allowing a 200 nm–20 μm useful optical range. The working distance is 10.4 mm with a numerical aperture (NA) of 0.52, an optical clearance of 5.6 mm, and an optical obscuration of 17%.

The beam is focused on the sample fixed to the piezoelectric-motor based stage XYZS. We are currently dithering the sample for greater ease of allocation of many different fixtures. The XY stage is a double M-664 stage (Physik Instrumente, Karlsruhe, Germany) with a 25 mm available range, 100 nm resolution, 200 nm unidirectional repeatability and a maximum speed of 400 mm/s. Under the XY stage, the Z movement is supplied by a Newport GTS30V stage that offers a 30 mm excursion with ±100 nm bidirectional repeatability and 100 mm/s maximum speed.

The specular reflection is then converted again to a parallel beam (of course, only if the sample is exactly at the focus point) and conveyed by a second relay group of plane mirrors (RL2) to the dichroic beam splitting and focusing group (B&F).

The second beam splitter (BS2) is a 2″ diameter 300 μm thick Silicon wafer, with both faces polished. This component allows a good reflection of the visible (VIS) part of the spectrum above the silicon gap. Instead, all the infrared (IR) part of the spectrum is transmitted through the silicon lamina for energies below the gap, starting around the Nd:YAG 1.064 μm seed line of the supercontinuum laser. On both sides of the silicon beam splitter, two off-axis parabolic mirrors (Newport 50338AL) focus the parallel beam onto two pinholes (PH1 and PH2) at the entrance points of two different spectrometers, making simultaneously available the VIS and the IR spectral regions. The parabolic mirrors have a focal distance of 10 cm, and with their magnesium-fluoride-protected aluminum coating can be used without chromatic aberrations between 200 nm and 10 μm. While in the past we have used variable sized pinholes, the complexity of the alignment with the spectrometers optics made us opt for a fixed size pinhole of 25 μm diameter. In the future, more flexible solutions could be available.

The spectrometers SPCVIS and SPCIR are the final points of the two split optical paths. The VIS spectrometer consists in a PIXIS CCD detector (Princeton Instruments, Trenton, NJ, USA) mounted on a SP2150 f/4 spectrograph (Acton, Princeton Instruments, Trenton, NJ, USA) equipped with two gratings of 1,200 L/mm and 150 L/mm, both with 500 nm blaze; the high resolution grating covers about 100 nm dispersed on the CCD, with a maximum resolution of 0.074 nm, while, with the other, we can use the full VIS range, dispersing about 1,000 nm on the detector, with 0.8 nm of spectral resolution. The detector used is a thermoelectric Peltier cooled (−80 °C) back-illuminated CCD, with 1,340 × 100 pixels, each 20 μm × 20 μm wide. The peak quantum efficiency is more than 95% between 500 nm and 650 nm, and the readout noise is of the order of 10 e- rms, making the detector very sensitive also for more demanding tasks than reflectance.

The IR spectrometer is realized by coupling a Newport MS127i imaging spectrograph with one of the InGaAs array detectors by Hamamatsu (Hamamatsu City, Japan), that is G9211-256S and G9208-256W 256 pixels each, with spectral ranges sensitivities between 0.9 μm to 1.67 μm and between 0.9 μm to 2.5 μm, respectively. Both sensors have a peak sensitivity of more than 1 A/W and an associate dark current of the order of 2 pA and 500 pA, respectively, making possible direct measurement of photon intensity in the nW range. The measurement that we report here has been obtained with the first of the two detectors. We are using a grating (75 L/mm, 1.6 μm blaze) to cover all the available range at once. The resolution is, in this case, 4.85 nm/pixel.

The controlling software, not shown here, has been written using custom C++ programming routines using the software drivers and libraries of all available instruments, realizing a virtual panel. Data are packed as 16 bit binary data, incorporated in a four-dimensional matrix that can be read cutting the hypercube along any possible interesting cutting plane.

Electronic signals are used to enable hardware synchronization of all different subsections, for instance to store, exactly at the same time, data from different detectors for the VIS and IR parts. The acquisition speed is determined by the limits imposed by both the array detectors, *i.e.*, about 1,000 spectra/sec, with VIS and IR parts acquired simultaneously in the own buffer, and takes into account the high precision of 16 bit/pixel for both detectors. Reducing the precision, and using other types of detectors, the speed could be substantially increased. Data are downloaded from both detectors after every line, being the scanning between the single pixels completely governed by hardware triggering among the instruments.

Each spectral point is loaded into detectors by means of a master electronic trigger generated by an optical chopper for every image pixel. As usual for spectroscopy, the chopper is also used to make the correct sequence of light and dark on detectors, cleaning charges between subsequent acquisitions. More recently, a virtual chopper has been used, introducing several ghost acquisitions collected at the beginning of each row and then discarded, in order to clean the detector's channels as well. Thus, in our current configuration the master electronic trigger is generated by the PI C867 260 XY motors driver producing transistor-transistor logic (TTL) triggers at specific calibrated points during the fast axis scan.

### 2.1. Optical Considerations

The distinguishing feature of this apparatus is the ability to collect reflectivity with the assurance that, for all the accessible wavelengths, the single spectrum associated with a physical pixel can be related with a single point with space coordinates X, Y and Z. Moreover, the optical performances are to be not too far from the typical characteristics of a standard reflectance confocal microscope. Therefore, some considerations can be usefully drawn at this point. In any case, the only very critical point is to avoid the chromatic aberration of the focus on the sample, after which any possible residual chromatism on the return path, being part of the transfer function of the microscope, can be incorporated in data normalization.

#### 2.1.1. The Beam Splitter

The beam splitter (BS1 in Figure 1) is the only part of the instrument not realized as a pure reflective component, so extreme care has been taken to minimize any possible chromatic aberrations. By design, the beam reaches the sample after reflection on the beam splitter, with the result that also the overall intensity flux on the sample surface has been considered for the best choice of this component. In fact, while the overall intensity on the detector side is the product of the beam splitter reflection and transmission, therefore irrespective of the order, the dose on the sample is not. In Table 1 below, a comparison between different solutions is given. A BK7 lamina has been adopted due to the fact that we often work with live cells, thus minimizing the irradiated dose on the sample. Other possibilities exist that can, instead, maximize the total through output.

Various adverse effects on the beam propagation are coming from multiple reflections as a function of lamina thickness and composition like: (i) a shift of the focus, due to a ghost filling of the objective input; (ii) a further shift that, after transmission, can cause an axial error on the return optical axis; (iii) a chromatic behavior that accompanies the two previous effects with an explicit dependence on the wavelength due to the material dispersion; (iv) interference effects within the slab that make the reflectivity measurement ill characterized. All those effects gain some importance because of the huge spectral range involved here, while they are almost negligible when dealing with a fixed frequency laser confocal microscope.

Upon hitting the beam splitter, and following the notation of Born and Wolf [19], the beam makes one reflection *r*_12_ on the first interface air-material and, then, a second reflection *r*_21_ between the material and the air. This second reflection is then refracted back, and comes out parallel to the first one, but shifted. The shift happens to be dependent on the slab thickness and on its index of refraction. In case of dispersion, such a shift displays a color composition on the beam side, instead of an otherwise white beam.

**Figure 2.**
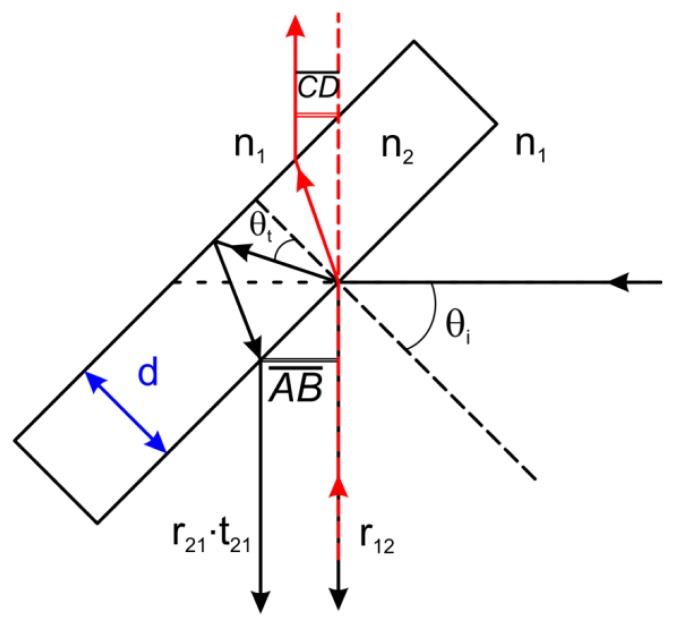
Optical paths of the reflected and the refracted rays on the first beam splitter.

Following the construction of Figure 2, that obviously represents only the main rays that we are considering, the amount of the shift *A̅B̅* results to be:

(1) $$\bar{AB}=2d\tan(\theta_{t})\cos(\theta_{i})$$

where the refraction angle *θ_t_* is related to the index of refraction of the material by the Snell's law. Some possibilities that we have explored are shown in Table 1. Two materials, both very well known for their wide IR transparency (down to 10 μm), *i.e.*, calcium fluoride and a β form of zinc sulphide (CLEARTRAN™ by CVD, Dow Chemical Company, Midland, MI, USA), cause huge shifts with non-negligible chromatic aberration. To move away the second reflection, a thicker CaF_2_ could be used, e.g., of about 10 mm, in order to extend the usability of the microscope farther in the IR, while for CLEARTRAN™ the required thickness is considerably more and not realistic. As it is clear, the thin BK7 lamina introduces a rigid shift *A̅B̅* of the order of 100 μm with a chromatic component of the order of 1 μm, that is absolutely negligible in relation to the 5.5 mm beam diameter, and has a transparency perfectly compatible with the current spectroscopic range of the microscope. In a similar way, it is possible to compute the shift *C̅D̅* of the output beam, considering only the reflection, and then the refraction of the main beam of amplitude *r*_12_:

(2) $$\bar{CD}=d\frac{\sin(\theta_{i}-\theta_{t})}{\cos(\theta_{t})}.$$

Again, the BK7 solution has a very good performance, with a very small chromatic aberration, while, generally, the magnitude effect is more dependent on the absolute value of the index of refraction.

The exact solution of the beam splitter reflectivity and transmittance takes into account multiple reflections, yet it can be expressed [19] in terms of *r*_12_ and *r*_21_:

(3) $$R=\frac{r_{12}^{2}+r_{21}^{2}+2r_{12}^{2}r_{21}^{2}\cos(2\beta )}{1+r_{12}^{2}r_{21}^{2}+2r_{12}^{2}r_{21}^{2}\cos(2\beta )},T=1-R,\beta =\frac{2\pi }{\lambda }n_{2}d\cos(\theta_{t}).$$

Using Equation (3) it is possible to evaluate the remaining quantities in Table 1, in particular the exact computation for BK7 sample irradiation, the overall beam splitter efficiency, and the residual interference pattern integrated over the spectral resolution: this pattern can be arbitrarily decreased by binning the detector channels, reducing spectral resolution. For other materials, for which the thickness rules out interference effects, the calculations merely report the corresponding Fresnel equations considering only the first reflective surface, because a realistic computation should evaluate the exact part of the shifted beam that eventually enters into the objective. Here, only the average between transverse electric (TE) and transverse magnetic (TM) radiations is considered. Polarization effects are indeed quite strong: for BK7 the ratio of TE polarization with respect to TM is about 10:1.

#### 2.1.2. The Reflective Objective

The use of reflective objective in microscopy is well consolidated whenever infrared [20] or special solutions [21] are required. The main characteristics we are interested in, with the adoption of a reflective objective, are the absence of any chromatic aberration, the perfect spherical aberration cancellation, because of infinite conjugate points [20], and a useful large working distance. Many FTIR commercial microscopes include this type of objectives, deserving detailed studies on their performances [22]. Usually, the lateral resolution measurement has to be compared with the theoretical point spread function (PSF). This is indeed not a trivial task, unless one adopts crude approximations or makes more detailed considerations [23]. Here, we only want to compare the experimental resolution with the expected one, even if with some approximation.

**Figure 3.**
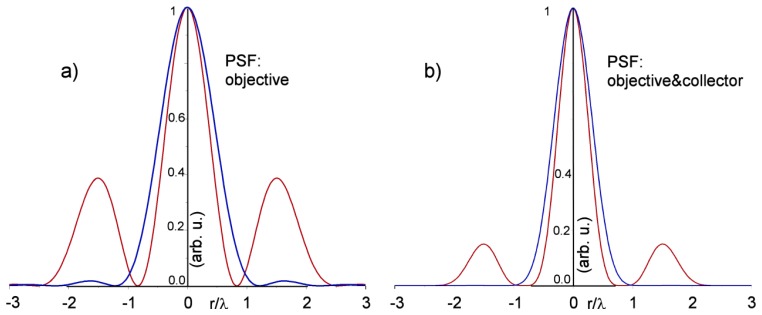
(**a**) Calculated PSF profile of a 0.52 NA objective (blue line) and a 0.52 NA annular aperture objective with 17% central obscuration; (**b**) Calculated PSF profile of the same objective as in (a) followed by the collecting lens (coinciding with the same objective). Normalized radial units are used.

The lateral resolution can be estimated by directly computing the corresponding annular aperture diffraction, coinciding with PSF if no aberration is included. We therefore write the wave amplitude at focus as:

(4) $$A_{\text{focus}}\approx \frac{J_{1}(2\pi \text{NAr})}{r}-\frac{J_{1}(2\pi \sqrt{\text{OBS}}\text{NAr})}{r}$$

where the arguments of the first order Bessel functions are: the Numerical Aperture NA, a radial coordinate normalized with the wavelength, *r*, and the relative radius reduction of the useful area given as the square root of the obscuration area (OBS). The expression is somewhat similar to Equation (4) of reference [23] that, however, seems wrong because of a parameter is placed outside the function argument, instead of inside. The focus intensity is shown for this objective with *NA* = 0.52 and obscuration area of 17% in Figure 3a, compared with a standard objective.

Figure 3b presents the intensity after passing the collector, *i.e.*, the same objective. The curves are very similar to well-known results [24], for which an annular objective produces an increase of the side lobes, also if the central one narrowing promises a better resolution than standard objectives. At the end, the lateral resolution computed for our NA is apparently slightly better, passing from the Airy radius *Airy* ≈ 0.61*λ*/*NA*=1.22*λ* to about 0.8*λ*. However, the contrast of the image can be lower, because of the side lobes, and can be better evaluated by the modulation transfer function (MTF), notoriously worse for this type of objectives.

#### 2.1.3. The Pinhole

The ability to discriminate the out-of-focus signal, typical of the confocal layout, is realized by focusing the returning beam on a pinhole. To retain some flexibility in the mechanical design, we realize the focus by using parabolic mirrors that rotate the beam by 90° without chromatic or spherical aberrations.

**Figure 4.**
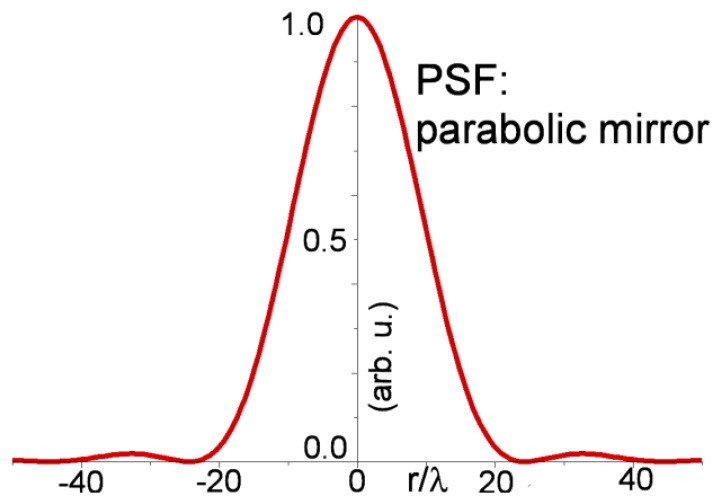
PSF focus radial intensity profile of parabolic mirrors expected from construction parameters. Normalized radial units are used.

**Figure 5.**
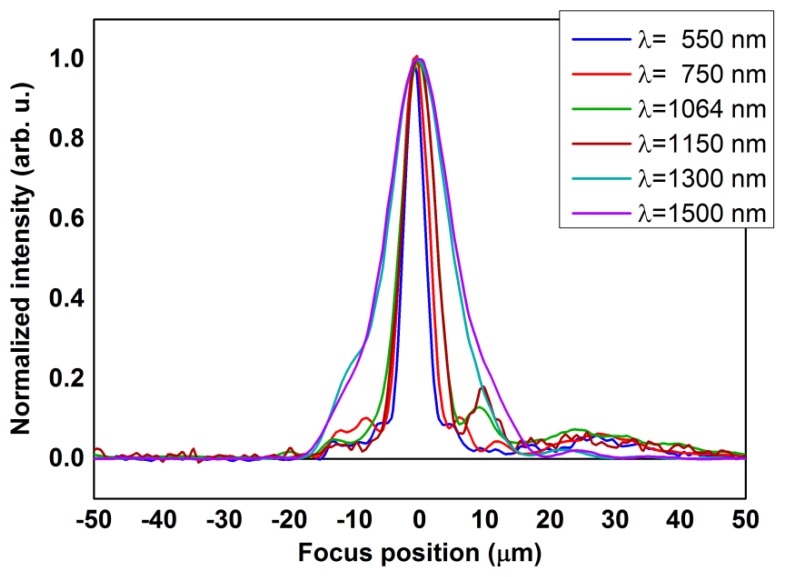
Reflectance intensity profiles at different wavelengths as a function of focus axial positions.

Due to the focal length and the beam diameter, the parabolic mirrors are equivalent to an optical lens with NA = 0.025. Below, in Figure 4, the produced focus is represented by an Airy intensity plot with an adimensional diameter of *r* ≈ 49, measured between the two first minima. With a pinhole diameter of 25 μm, the focus completely enters the pinhole only at the wavelengths around 0.5 μm, being cropped at longer wavelengths. This type of chromatic aberration is only relevant for the signal intensity as measured by the detectors, but has no consequences on the achromaticity, as it is possible to deduce by the axial measurements made to evaluate the out of focus discrimination (see Figure 5).

## 3. Results and Discussion

In the following paragraphs, we present experimental results useful to define both the axial resolution and the lateral one. As explained in Section 2, we have realized a system that manages a rather large spectral range so to obtain performances related only to physical quantities, such as the specific wavelength, and not to accidental factors, like for instance, the difficulty to maintain exactly focused two different spectrometers that work with two different detectors in completely different spectral ranges.

It is easy to show the usefulness of this hyperspectral confocal microscope for solid state and other technological application. Here, we present the simple case of the silicon/silicon oxide calibration sample, where the explicit dependence of the local reflectivity on chemically different areas allows precise measurements of the local thickness. This example would be used as a template for a vast range of problems, mainly of a technological kind, for which local composition and local properties are of some value for a deep analysis of the sample properties.

The original purpose of the realized microscope is its application to the study of biological matter, like living cells or tissues. In this case, the large accessible spectral range can increase the inventory of label marking, without adding exogenous compounds, via only the spectral features of the specific biological state of the particular specimen. The final goal of a label-free spectroscopic fingerprinting has to be still fully demonstrated mainly because of lacking of clear biochemical patterns in the near IR spectral region. However, we show here that such a purpose can be fruitfully addressed by using statistical methods for data classification, as usual for hyper-spectroscopic results.

### 3.1. Axial Resolution

The simplest and common way to evaluate the axial resolution of a confocal microscope is to measure the output intensity reflected by a mirror surface as a function of an axial movement of the sample or the objective along the Z axis. This quantity is directly related to the out-of-focus discriminating ability, although less clearly defined when the beam focus is immersed in a multilayered or highly scattering medium. A not exhaustive literature exists for reflective objectives, the obtained results are nevertheless not very far from a classical formulation.

For this purpose we have used a really flat silicon sample covered by a 1 μm thick silicon nitride layer, coated with 100 nm of chromium and 100 nm of gold, with an overall roughness of few nanometers, with engraved a micrometric square. This last structure is used below for lateral resolution measurements, while the mirror surface is used for axial resolution.

Figure 5 shows some selected wavelengths within more than one thousands collected wavelengths.

The FWHMs of the shown curves have a value of 3.93 μm at a wavelength of 0.55 μm, 6.51 μm at 1.064 μm, reaching 11.16 μm as measured at a wavelength of 1.5 μm. We can compare the experimental results with the expression elaborated for wide field microscopy [25] and suitable for standard objectives:

(5) $$z_{\min}≃\frac{2\lambda }{{\left(NA\right)}^{2}}.$$

The expression, valid in air, represents the distance between minima in a reasonable description of the axial focus. Taking into account that *NA* = 0.52, the measured values obey Equation (5) within ±5% on average. Therefore, despite the crude approximations, the axial resolution is aligned to the predictable performances of our microscope based on the optical properties of the used objective.

### 3.2. Lateral Resolution

**Figure 6.**
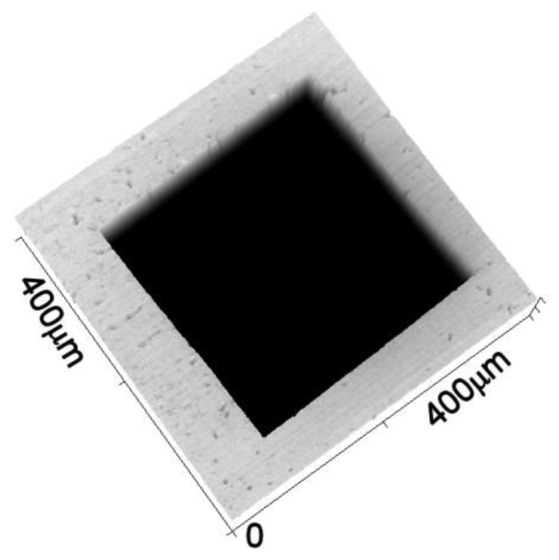
Image at 550 nm wavelength of square hole in Au coated silicon nitride membrane. The lateral side of the image is 400 μm.

We have evaluated the lateral resolution by imaging the stepped edges of a square hole realized on the sample referred in the previous section. The method has the advantage of simplicity: acquiring the image, and selecting one or more sectioning profiles, we simultaneously obtain a profile for all accessible wavelengths. In Figure 6 we show the entire structure within a 400 μm wide image obtained with our microscope, selecting a wavelength of 550 nm.

We only measure the optical intensity, but we know that the surface roughness is very small, of the order of few nanometer, and that the Electron Beam Lithography (EBL) used to realize this structure is able to produce a sharpness of a few tens of nanometers, also including the chemical processes that follow the EBL part of work. Therefore, the measured step width is broadened essentially by the optical resolution, with the addition of the stage motion inaccuracies. The measured resolution is thus an upper limit that includes mechanical errors that, on the already given specifications of our stage, are likely to be not so important.

**Figure 7.**
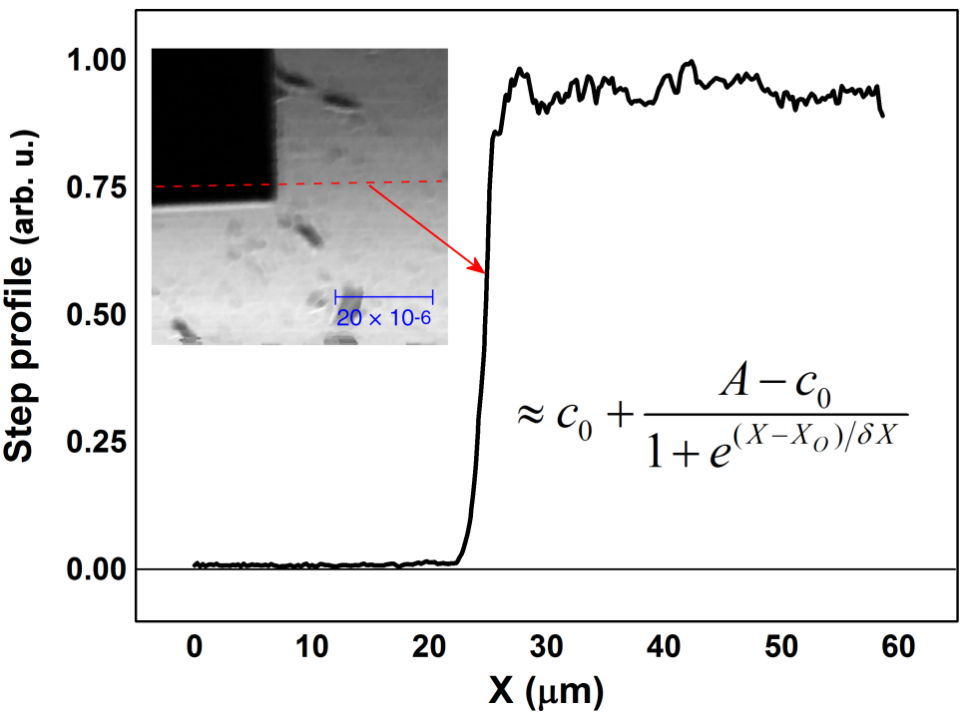
Reflected intensity measured on the edge of the square hole. The extraction region is indicated by the dashed line on the inset image (wavelength 550 nm, lateral size 60 μm).

A normalized step profile is visible in Figure 7. Embedded in the figure is reported the 60 μm wide image, again selected at 550 nm and with a pixel resolution of about 230 nm, used to extract the profiles.

We make a best fit of the measured profile using a Boltzmann-like step function:

(6) $$I_{\text{pro}}\approx c_{0}+\frac{A-c_{0}}{1+e^{\left(X-X_{O}\right)/\delta X}}.$$

**Figure 8.**
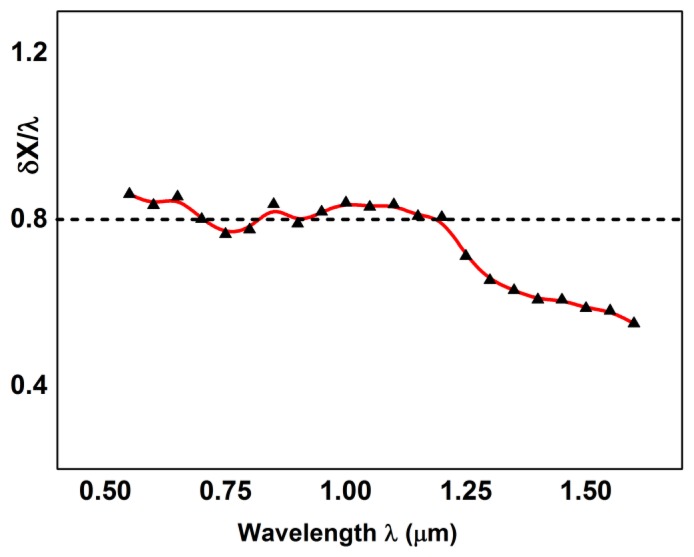
Fit of the lateral resolution, normalized to the wavelength, and plotted as a function of the wavelength.

Following Equation (6), *c_o_* is the intensity offset, very near to zero, *A* is the step function amplitude, and the *δX*, that widens the step around the pivot point *X_O_*, assumes the role of lateral resolution [18]. It is possible to compute the *δX* parameters from the results obtained at different wavelengths. Moreover, we know that the theoretical lateral resolution can be written as *δX_Airy_* ≈ *k λ*/*NA*. Usually, the parameter *k* is 0.61, in the case of a pure Airy function and, because in our case NA is nearly 0.5, the expected value is *δX_Airy_* ≈ 1.22*λ*. Therefore, it is meaningful to plot the quantity *δX*_exp_/*λ* to check the experimental results against the theoretical values. The final result is visible in Figure 8. It is clear that the trend is very different compared to the value of 1.22, but is very near to the value of 0.8 that we have deduced from the theoretical PSF of our objective. It means that, technically, we are working in a super-resolution regime. However, the optical performances are also related to the contrast expressed by the PSF lateral lobes weight, as already explained. Nevertheless, whenever a sample is imaged with intrinsic high contrast, e.g., technological samples, reflective objectives are able to perform better than more usual objectives.

### 3.3. Spectroscopy on Semiconductor Samples

The most obvious application of our microscope is the measure of reflectivity. The advantages of this method are various and innovative. Reflectivity is measured on a local scale, close only to the beam focus surroundings. It can be measured on an buried interface or a discontinuity, and not exclusively on the sample surface: this requires the specimen transparency but, due to the available spectral range, it is possible to reach the hidden face by choosing the right wavelengths; as an example, one can image the buried interface of evaporated layers on a semiconductor substrate simply going below the energy gap of the material and imaging from the back-side of the device [18].

Moreover, the accessible spectral range is often the region of strong variation of the optical constants for the most common materials, so very precise measurements of the composition or thicknesses of a sample can be derived from experimental data. The sensitivity of the method is such that differences of few nanometers in the thickness of the observed layers can be readily observed, and an example follows.

**Figure 9.**
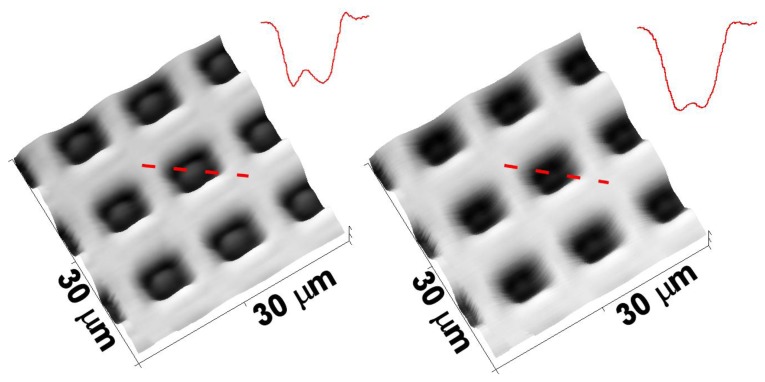
AFM Calibration sample image at: (**a**) *λ* = 550 nm; (**b**) *λ* = 650 nm. The lateral side of the images is 30 μm. The measured intensities profiles are indicated by the dashed lines.

**Figure 10.**
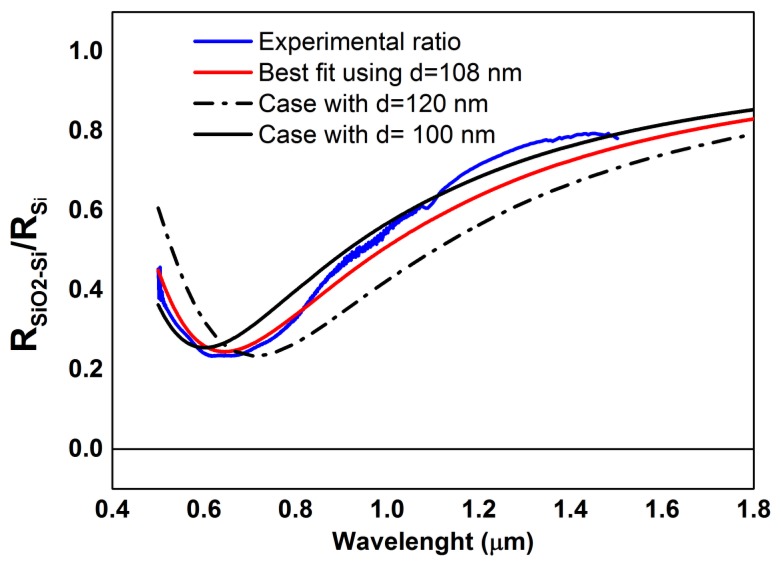
Silicon oxide/silicon reflectance ratio as function of wavelength. Experimental (blue line) and calculated trend for different thickness of silicon oxide layer: 108 nm (red line), 100 nm (black line), 120 nm (dotted line).

We have used an Atomic Force Microscopy (AFM) calibration sample with periodic structures of SiO_2_ on a silicon substrate (Nanosurf^®^ AG, Liestal, Switzerland). The silicon oxide squares have a side of 5 μm with a periodicity of 10 μm. The images of the structure in Figure 9 clearly show the spectroscopic effect. In fact, while in the image layer at *λ* = 550 nm (left) some protrusions in the black square structures are visible, in the image at *λ* = 650 nm (right) the squares appear as deep minima, as clarified by the profile inserts: data are dominated by spectroscopic effects, a consideration that is not taken in the proper consideration when images are obtained with standard, single wavelength, confocal microscopes. To understand this effect it is worthwhile to underline that it is quite difficult to measure the absolute reflectivity, because an absolute reference inserted in the same optical path should be available. Instead, it is possible to experimentally evaluate the ratio between two compositionally different sample zones, in this case the silicon oxide respect to the bare silicon. The experimental ratio, averaged over contiguous square zones, is shown in Figure 10.

The experimental data (blue line) are reproduced quite well by the theoretical calculation (red line) for a thickness of the silicon oxide hill of 108 nm, against a declared value of 100 nm and AFM measured value of 119 nm, referred to the top maximum of the structure. The theoretical lines are computed by a full multilayer calculation [18] based on the Abelés matrix method [19]. Dielectric functions for silicon and silicon oxide are taken from Palik [26]. The sensitivity of this methodology can be appreciated by noting that theoretical calculations for the thicknesses of 100 nm (black line) and 120 nm (dotted line) are clearly incorrect.

### 3.4. Biological Samples; Statistical Representation

The reflectance hyperspectral confocal microscope described so far has been developed in the framework of a national project aimed at performing optical spectroscopy of the skin in three dimensions in order to assess the possibility of carrying out diagnosis and classification of skin pathologies using optical spectroscopy. In this context, the investigation of biological specimens represents a primary objective. We show an example of discrimination of cellular compartments based on spectral features, achieved with a principal component analysis on hyperspectral data.

We have used M101221 melanoma cell line derived from metastasis recurrence in a patient during vemurafenib therapy treatment, kindly provided by Prof. Dummer (University Hospital, Zurich, Switzerland). Cells were grown in RPMI-1640 medium supplemented with 10% (v/v) inactivated Fetal Bovine Serum, 1% penicillin/streptomycin (medium, serum and antibiotics purchased from Lonza, Basel, Switzerland) and 1% Na pyruvate (Life technologies, Carlsbad, CA, USA). Cells were maintained at 37 °C in a humidified atmosphere of 5% CO_2_ and 95% air until observation.

**Figure 11.**
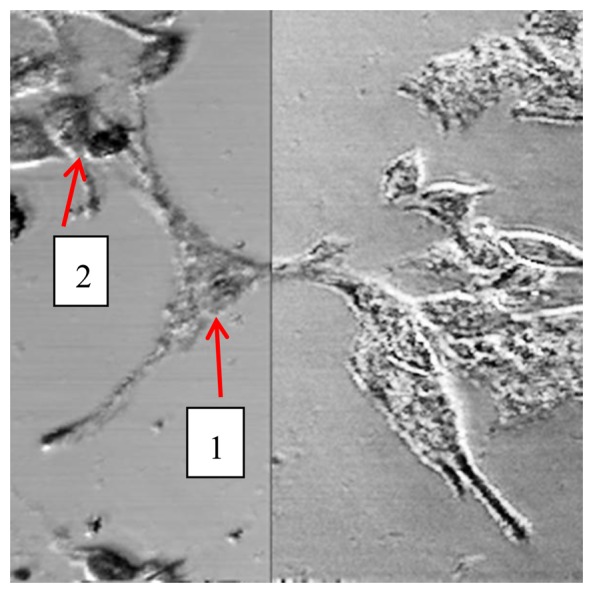
Image of human melanoma cells. In the left half the image at 1,064 nm wavelength, in the right half at 550 nm. Lateral dimensions 400 μm. Cells considered for analysis are indicated by arrows.

To perform measurements on melanoma cell culture, we used a culture chamber specially designed and 3D printed (HP Designjet 3D printer, Hewlett-Packard, Palo Alto, CA, USA) in our laboratory. Cells have been plated and cultured on 40 mm diameter round coverglass in a 60 mm Petri dish. 24 h after seeding, the coverglass was mounted as bottom of the chamber, sealed with a previously sterilized 2 mm thick silicone gasket. The chamber has then been fixed on the microscope stage to perform acquisition. Considering laser power emission, focusing optical system characteristics and culture chamber parameters, we estimated the light power on the cell layer to be less than 4 μW/nm. Extensive light exposure has been performed in different cell cultures with no evident damage. Figure 11 shows an image of melanoma cells (400 μm × 400 μm) at λ = 1,064 nm (left portion) and λ = 550 nm (right portion) to highlight how the availability of spectral images allows to visualize different features also in biological samples.

Spectra from nuclear areas and from cytoplasmic-membrane areas of two different cells (arrows) have been acquired and averaged over a circular region of interest (23 μm diameter). As internal reference we have used a portion of glass substrate free of cells: every spectrum has been normalized to the reference spectrum. For each cell, average spectra from two different nuclear areas have been acquired. Four spectra from cytoplasmic areas in the cell labeled 1, and two spectra from cytoplasmic areas in the cell labeled 2, have been acquired as well. To visualize images and extract average spectra from the regions of interest we have used custom-developed software.

**Figure 12.**
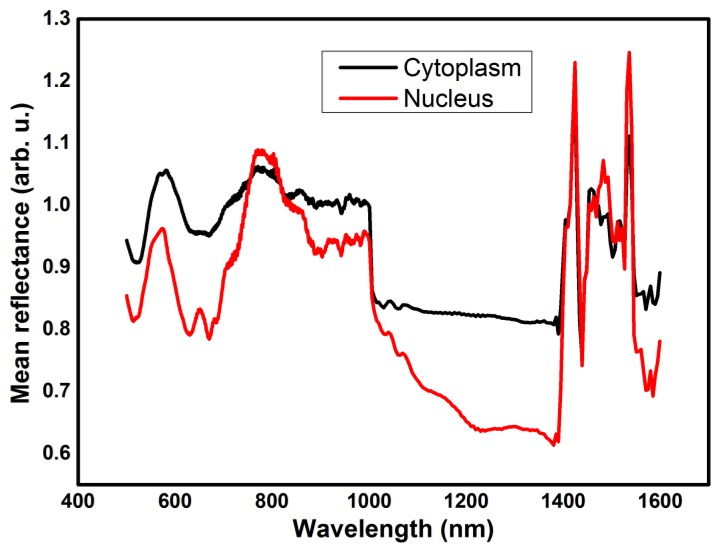
Mean reflectance spectra of cytoplasmic areas (black) and nuclear areas (red) from melanoma cells. Each spectrum is the result of the sum of visible spectrum (500 nm–1,000 nm) data from visible sensor and infrared spectrum (1,000 nm–1,600 nm) data from IR sensor.

From the spectra in Figure 12 it is evident that both in the VIS and in the IR regions there are spectral features that may lead to the identification of different compartments. However, it must be considered that the spectral response we obtain is the combination of a morphological contribution from scattering effects and a chemical contribution from absorption effects. For this reason, especially dealing with complex samples, a pure light scattering interpretative model [27] or a pure chemometric model may be reductive.

Recent advances in analysis instrumentation and in data collection techniques have resulted in a rapid increase in the amount of data acquired from spectral imaging. Extracting the significant information from the data produced by modern instrumentation is, in many circumstances, a nontrivial task. Here we introduce the application of statistical tools to sample analysis and modeling. These tools can be used to extract useful indications about the structure and the nature of the sample, leading to highly reliable classification.

**Figure 13.**
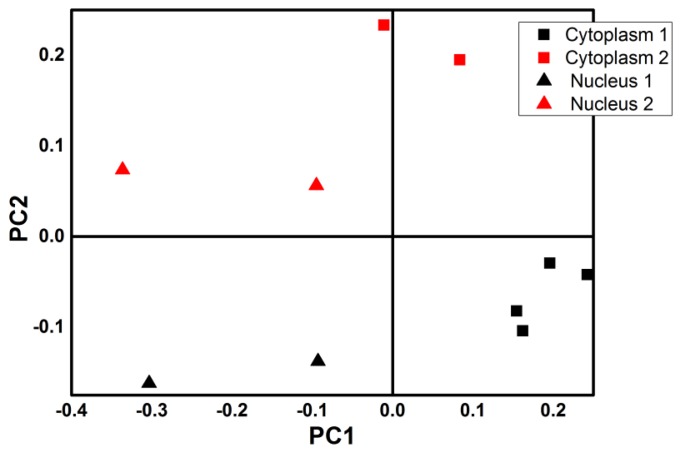
Score plot of PCA analysis on cellular compartments of melanoma cells in PC space. The first two components have been considered: points represent spectra from cell1 (black) and from cell2 (red), from nuclear areas (up triangle) or from cytoplasm (square).

Previously described spectra from nuclear and cytoplasmic-membrane areas of two different cells have been considered for principal component analysis (PCA). Average spectra from different areas have been smoothed with a Savitzky-Golay algorithm and compared using PCA. Normalized reflectance intensity at nine different wavelengths, spanning from 500 nm up to 1,300 nm (with 100 nm interval between wavelengths) have been considered as variables. The first two principal components have been considered (cumulative variance 87.12%) and points representative of average spectra from different areas and different cells have been plotted in a PC1/PC2 space. Then different shapes have been assigned to different compartments, and different colors have been assigned to different cells (Figure 13).

From the plot in Figure 13 it is easy to recognize that spectra from different cell compartments can be separated, for they occupy different half planes of the PC1 dimension (except one point), but spectra from different cells can also be distinguished for they occupy a different half plane of the PC2 dimension. This analysis usually represents the first step of more complex classification and clusterization methods. Within this scope, the application of a multivariate approach to hyperspectral data, although not new in remote sensing and food/drug analysis [28–30] and coupled to FTIR and Raman micro-spectroscopy [31], as far as we know, has not been used in reflectance confocal microscopy with cell culture. It is important to underline that PCA is an unsupervised method and that the qualitative results are independent from the specific set of sampling wavelengths, thus making it a valuable and adjustable tool to perform first analysis in different frameworks.

## 4. Conclusions

The new confocal microscope presented here has been realized with the idea of integrating structural and morphological information with detailed spatially resolved spectroscopic properties. Every pixel in the obtained confocal image is associated with spectral information in the wide range allowed by the combined characteristics of the laser source and the two detectors for radiation reflected by the sample (0.5 μm–2.4 μm wavelengths).

Consequently, this technique is an attractive tool for a wide range of applications in many critical areas, including bioscience and medical diagnostics, inorganic and organic material sciences, environmental sciences, forensics and archeology. Data are acquired with sub-micrometer spatial resolution and high spectral resolution. Preliminary data with clear different cells discrimination show that also if reflectance has no specific sensitivity to biochemical properties, a spectroscopic classification is possible. Optical differences between different cells can be attributed to real chemical compositional differences or to a mere variation in the density of the same constituents, contributing anyway to the index of refraction local definition and to very sensitive measurements.

## Figures and Tables

**Table 1.** First beam splitter performances for various materials as a function of the wavelength λ. All distances are given in mm, while the irradiation and efficiency are given as percentage of the incoming beam, as well as the pk–pk amplitude of the interference effect. Here the values are averaged taking into account the spectral resolution as given in Section 2.1.

**Beam Splitter Material**	**d**	**Beam Shift (*A̅B̅*) at λ: 0.5 μm, 1 μm, 1.5 μm, 2 μm, 2.5 μm**	**Beam Shift (*C̅D̅*) at λ: 0.5 μm, 1 μm, 1.5 μm, 2 μm, 2.5 μm**	**Sample Irradiation, Overall Efficiency**	**Interference Amplitude**
CaF_2_	6	4.8, 4.83, 4.85, 4.86, 4.87	1.84, 1.83, 1.82, 1.81, 1.81	4.1% 3.8%	-
CLEARTRAN™	6	2.65, 2.78, 2.79, 2.79, 2.80	2.92, 2.85, 2.85, 2.85, 2.84	16% 13%	-
BK7	0.16	0.119, 0.12, 0.121, 0.122, 0.122	0.054, 0.053, 0.053, 0.052, 0.052	9.3% 7.8%	0.2%
